# Advanced Practice Nursing Development Within Diverse Regulatory Landscapes: A Descriptive Survey of European Countries

**DOI:** 10.1111/inr.70250

**Published:** 2026-10-12

**Authors:** Caroline Bohlender, Karen Bjøro, Paul Gill, John Unsworth

**Affiliations:** ^1^ School of Healthcare and Nursing Sciences Northumbria University, Newcastle‐upon‐Tyne UK; ^2^ The European Forum of National Nursing and Midwifery Associations Oslo Norway; ^3^ Nursing and Head of School of Healthcare and Nursing Sciences Northumbria University, Newcastle‐upon‐Tyne UK; ^4^ Community Nursing and Workforce Policy Centre for Health and Social Equity Northumbria University, Newcastle‐upon‐Tyne UK

**Keywords:** advanced practice nursing, Europe, professional regulation, scope of practice

## Abstract

**Aim:**

To explore the relationship between professional regulatory systems and advanced practice nursing development in Europe.

**Background:**

Advanced practice nursing has expanded rapidly, yet regulatory frameworks have often struggled to keep pace with the extended scope of practice demanded by contemporary service delivery. Misalignment leaves new roles inadequately supported, with consequences for both patient safety and the broader transformative potential of the profession.

**Methods:**

For this cross‐sectional descriptive study, key informants were recruited by purposive sampling across the World Health Organization's Europe Office member countries, including regulators, educators and nursing association representatives. A purpose‐built questionnaire was analysed using descriptive statistics and pattern comparison.

**Results:**

The majority of European countries have advanced practice nursing roles, yet scope of practice and accompanying regulatory mechanisms varied considerably. Independent prescribing authority was the least commonly reported characteristic, having been established in only four countries. An observed descriptive pattern indicated that role maturity was related to government‐led or statutory regulation designed specifically to address extended clinical skills. Interest‐holders were largely in favour of regulatory intervention.

**Conclusion:**

Regulatory modernisation is a key step for health systems seeking to expand advanced practice nursing. Legal scopes of practice must be realigned with clinical realities. In this sample, reported independent prescribing authority co ‐occurred with government led or legally mandated regulatory arrangements.

**Implications for Nursing:**

Nursing organisations should advocate for mandatory frameworks encompassing title protection, standardised educational requirements and competency standards for high‐risk clinical skills, such as independent diagnosis and prescribing.

**Implications for Health Policy:**

Governments should establish statutory advanced practice frameworks, with nursing leadership within government playing a key role.

## 1 Introduction

Globally, health systems are contending with increasingly complex epidemiological profiles, persistent workforce shortages and escalating pressures to deliver accessible and high‐quality health services. Advanced practice nursing (APN) has emerged as a key strategy for strengthening healthcare provision by expanding clinical responsibilities beyond traditional professional boundaries (World Health Organization 2021). However, these roles should be effectively integrated into health structures to increase service delivery and improve patient outcomes.

As advanced practice roles develop and clinical responsibilities shift, variability in educational preparation, inconsistent practice and ambiguous accountability all pose risks to patient safety. Without robust regulatory frameworks, these risks move from theoretical to tangible (Giarelli and Saks 2023). Professional regulation has been widely recognised across the literature as a mechanism for managing these vulnerabilities, yet nursing regulatory organisations and mechanisms used remain highly diverse within the European region. Consequently, APN has, in effect, outgrown the regulatory structures designed for the broader profession.

For policymakers and healthcare providers, this represents both an opportunity and an obligation to harness the transformative potential of APN while ensuring its safe, effective and coherent integration into existing service structures. This article addresses a gap in the evidence base by exploring a potential relationship between regulatory conditions and APN development across the World Health Organization (WHO) European region.

## 2 Background

APN has firmly established itself as one of the most rapidly expanding segments of the global health workforce, with 53% of countries worldwide having introduced advanced practice roles by the first State of the World's Nursing report (World Health Organization 2020), rising to 75% just five years later (World Health Organization 2025). However, these figures mask considerable variation in what APN means across contexts, as a role title alone provides no indication of the scope of practice exercised or the regulatory conditions governing it.

The ICN APN Guidelines (International Council of Nurses 2020) provide a widely accepted international reference point, defining the complete expression of advanced practice nursing as the combination of an extended clinical skill set with nursing philosophy and full practice authority (FPA); the legal right to diagnose, prescribe and refer independently. This comprehensive incorporation of capabilities is consistently associated with the strongest improvements in health system performance (Htay and Whitehead 2021). Despite international consensus on its value, achieving FPA remains far from the global norm, frequently constrained by legally defined professional boundaries and entrenched organisational structures (Wheeler et al. 2022).

Five primary cross‐national studies have examined APN development, four including Europe within a global sample and one focused on Europe alone (De Raeve et al. 2023). Comparing findings across these studies is complicated by nomenclature difficulties and variability in how APN roles were defined and measured (Heale and Rieck Buckley 2015; Pulcini et al. 2010)—most strikingly illustrated by the fact that some countries identified as having APN roles in the earlier studies appear without them in De Raeve et al. (2023). Wheeler et al. (2022) observed that regulation was the most difficult aspect to capture and reliance on English‐only instruments may have further disadvantaged non‐English‐speaking respondents.

Across the literature, regulation is consistently identified as both a key enabler and a barrier to APN development. The WHO Global Strategic Directions for Nursing and Midwifery (World Health Organization 2021) identifies modern, competency‐based regulation as essential to enabling the nursing workforce to contribute fully to Universal Health Coverage, acting as both a facilitator of innovation and a mechanism for public safeguarding. Nevertheless, few studies have examined regulatory structures and APN development with sufficient granularity. Maier (2015) identified three broad regulatory models based on level and locus of governance, but did not systematically examine whether the type of regulatory organisation, governmental or professional, influenced outcomes. Though De Raeve et al. (2023) identified the bodies conferring APN recognition, the relationship between regulatory body type and APN development remained unexamined.

In many settings, APN roles have developed organically, shaped more by immediate service pressures than intentional system design, and in the absence of regulatory alignment, this ad hoc expansion introduces an unacceptable risk to patient safety (Palmer et al. 2023). This has fuelled opposition from medical professionals, who argue that inadequately governed role expansion compromises care quality; opposition that in turn slows progress towards formal recognition and FPA (Kleinpell et al. 2023).

European professional regulation compounds this challenge, remaining highly heterogeneous and often grounded in historical tradition rather than contemporary evidence. Whilst most national regulators share a public protection mandate, their structures and operational mechanisms differ markedly (Benton et al. 2013), and divergence is particularly pronounced at advanced practice level (McConnell et al. 2013). As health systems have evolved rapidly, regulatory frameworks in many European countries have not kept pace (Antão et al. 2023). The resulting proliferation of regulatory types and APN definitions makes meaningful comparison difficult, obscuring opportunities to identify best practice and derive transferable lessons. Coherent, contemporary regulatory frameworks are therefore not merely desirable; they are a strategic necessity if APN is to fulfil its potential across Europe.

## 3 Research Question

How do regulatory body type and associated mechanisms relate to the development of APN across Europe?

## 4 Methods

The study utilised a cross‐sectional survey. Data are reported in accordance with the STROBE (Strengthening the Reporting of Observational Studies in Epidemiology) checklist (von Elm et al. 2008).

The inclusion of 53 countries within the WHO Regional Office for Europe (WHO/Europe) was deemed necessary, as each is a signatory to the Strategic Roadmap for Nursing and Midwifery, thereby demonstrating a commitment to developing the healthcare workforce (World Health Organization 2021). The region is divided into six subregions: Central Asia, Eastern Europe, Northern Europe, Southern Europe, Western Asia and Western Europe (United Nations Statistics Division 2023). Purposive interest‐holder sampling targeted three key groups per country (regulators, educators, representatives of national nursing associations), offering the potential for multiple perspectives within each country and ensuring participants had a high level of nursing expertise, which maximised the likelihood of obtaining accurate, contextually grounded insights into national APN structures, regulatory mechanisms and role implementation.

Data were collected using a purpose‐built questionnaire (Supplementary File S1). The instrument contained three parts. Part 1 collected demographic data; gender was recorded to characterise representation in positions of professional influence and leadership; professional background to confirm nursing affiliation; and length of service as a proxy indicator of expert status. Part 2 asked participants to identify regulatory organisations and mechanisms used for entry to nursing, providing a professional regulatory baseline. Individual mechanisms, such as registration, title protection and revalidation, were itemised to reflect cross‐national variation in adoption. APN scope of practice was reported by respondents against the ICN APN Guidelines (2020), operationalised by six concrete clinical activities (medical history taking, physical examination, ordering diagnostic tests, prescribing, referral to other specialities, and admission and discharge). Specific APN regulatory measures were sought, mirroring the nursing regulatory questions. Part 3 explored interest‐holder opinions through a Likert scale. Items were developed through systematic, thematic collation of the APN regulation literature using NVivo, capturing contextual factors influencing APN development where core concepts such as autonomy, expertise and accountability resist universal operationalisation. Themes were reformulated to Likert‐compatible statements, which were then scored on a 5‐point scale: strongly agree, agree, neutral, disagree or strongly disagree. Content validity indexing was performed by a panel of ten expert reviewers drawn from the three interest‐holder groups targeted by the survey, seven of whom were non‐native English speakers, reflecting the linguistic diversity of the study population. The scale‐level content validity index (S‐CVI/Ave) was 0.89 for relevance and 0.87 for clarity, both exceeding the accepted threshold of 0.78 (Polit and Beck 2006). The instrument was piloted by the same review panel prior to full distribution.

The questionnaire was designed in English with forward and back translation by a native speaker into Russian, as both are the official languages of WHO/Europe. The questionnaire was available by email invitation via the Joint Information Systems Committee for UK Higher Education (JISC) platform and distributed between December 2023 and May 2024. National contacts were identified for each of the interest‐holder groups using existing networks and publicly available data sources. Questionnaires were distributed by email to 159 named contacts (53 countries × three interest‐holder groups per country).

Data analysis was performed using descriptive statistics for demographics and country coverage. Scope of practice characteristics and regulatory organisations were categorised and compared. In the event of interest‐holder disagreement, the majority response was used; where tied, free‐text comments were consulted or, where possible, verified directly. Only Portugal was tied: the national nursing association, which also serves as the country's regulator, was contacted directly and confirmed the absence of APN‐specific regulation.

The study gained ethical approval from Northumbria University ethics committee (registration number 3395 Ref no. 2023‐3395‐2808, date 22 May 2023). Participants were invited via email with an attached Participant Information Sheet which detailed that participation was voluntary and anonymous.

## 5 Results

### 5.1 Sample Characteristics

Responses were received from 43 of the 53 countries. The European subregions (Eastern, Northern, Southern and Western, *n* = 42 countries) had a 93% response rate (*n* = 39/42), missing only the microstates of Andorra, Monaco and San Marino, whose small size means they are often reliant on neighbouring countries for workforce and specialist care. Central Asia (*n* = 2/5) and Western Asia (*n* = 2/6) returned lower response rates of 40% and 33%, respectively. A total of 97 country/interest‐holder group responses were returned for analysis, representing 61% of the invitation total.

Interest‐holder groups had similar response rates, with 64% for educators (*n* = 34) and 66% for national nursing associations (*n* = 35). Regulatory bodies had a lower response rate, with 28 of the possible 53 responses (53%). Within the groups, most respondents were nurses (87%). Some regulators had other professional backgrounds: physicians (2%), lawyers (1%), public health specialists (1%), and others (9%). A total of 90% (*n* = 87) of respondents reported at least 10 years of professional experience, with the majority (*n* = 70, 72%) over 21 years. Twenty‐two per cent of respondents were male (*n* = 21), a proportion higher than typically observed within the nursing workforce. Representation of male experts varied geographically, with the highest share observed in Southern Europe, where 46% were male (*n* = 11).

### 5.2 How Nursing Is Regulated

Across the WHO/Europe region, nursing regulation is predominantly government‑led, with regulatory responsibilities sometimes shared between the Ministries of Education and Health. Organisational hybrid regulatory models were frequently reported, most commonly between government authorities and national nursing associations. Regional variation was reported; Serbian respondents indicated the absence of any formal nursing regulatory framework, although in Kosovo, regulatory mechanisms are exercised through their national nursing association. This study used WHO/Europe as its sampling frame (53 member states). Kosovo is not recognised as a WHO member state and is therefore not included separately in the country count. However, responses were received from healthcare professionals in Serbia and Kosovo; Kosovo has its own distinct APN policy context, separate from Serbia's. These responses are reported under Kosovo rather than Serbia, as attribution to Serbia would misrepresent the policy landscape in both territories.

Disagreement existed among interest‐holders regarding the regulatory body responsible for each mechanism; consequently, categorisation of regulatory typology was based on the majority opinion of national interest‐holders. Table 1 presents the categorisation of nursing regulators by country.

**TABLE 1:** Categorisation of nursing regulators of responding countries.

Regulatory body categories	Country
Government	Austria, Belarus, Belgium, Bosnia and Herzegovina, Czechia, Denmark, Estonia, Finland, Germany, Hungary, Iceland, Lithuania, Luxembourg, Netherlands, Norway, Russia, Sweden, Tajikistan, Turkey, Ukraine
Government and National Nursing Association	Croatia, Greece, Italy, Kyrgyzstan, Latvia, Moldova, Slovakia, Spain, Switzerland
National Nursing Association	Albania, Bulgaria, Cyprus, (Kosovo), North Macedonia, Poland, Portugal, Romania, Slovenia
Independent Statutory Body	France, Ireland, UK
Government and Independent Statutory Body	Malta
No regulation	Serbia
No answer to question	Montenegro

The most frequently reported regulatory mechanisms for entry to nursing practice included the setting of education standards, professional registration, title protection, definition of scope of practice, specialty or branch registration and registration revalidation. However, not all countries applied the full range of mechanisms, with substantial national variation observed. For example, Germany has national‑level regulation of education standards and title protection; other regulatory functions were at the federal level, causing variation, as only 2/16 Länder maintained a nursing register. Finland and the Netherlands have no formal recognition of nursing specialties or branches. Register revalidation processes were not universally implemented: 14 countries reported the absence of revalidation requirements, only maintaining qualification registers.

### 5.3 APN Presence and Maturity

Among the 43 responding countries, 67% (*n* = 29) reported the existence of APN roles. Of the 14 countries reporting no APN roles, 9 (Bosnia and Herzegovina, Bulgaria, Croatia, Greece, Italy, Latvia, Poland, Romania and Russia) indicated plans for future implementation.

While the overall presence of APN roles across Europe aligns with global trends (World Health Organization 2025), the more detailed breakdown of characteristics reveals considerable variation in role maturity. In some cases, interest‐holder disagreement on scope of practice persisted, highlighting the heterogeneity of role conceptualisation within individual countries. History taking and physical examination were the most commonly reported elements of scope of practice, whereas diagnostics and prescribing were less consistently established. Together, these four characteristics allow a complete episode of patient care to be independently managed; full interest‐holder agreement on prescribing authority consistently corresponded with full agreement on diagnostic authority, whereas countries where interest‐holders disagreed on these characteristics did not appear to grant APNs the authority to independently manage a complete episode of patient care (see Supplementary File S2 for full response breakdown).

Several country‐specific configurations illustrated the breadth of this variation. In Albania and Kosovo, APN roles were primarily framed around education, research and management rather than expanded clinical practice. Cyprus presented a different challenge: While interest‐holders reported that APN education covering all core competencies had been completed, regulatory barriers were delaying implementation, resulting in divergent interest‐holder responses depending on whether they were describing intended or current scope of practice.

Prescribing authority is an indicator of a comprehensive advanced practice model and is presented as a standalone classification (Table 2), given its centrality to independently managing a complete episode of care. Free‐text comments clarifying respondents’ binary answers allowed a more nuanced classification to be constructed: Independent prescribing authority was identified in four countries, with a further three showing constrained prescribing authority. These findings suggest that legal authority, professional autonomy and functional practice do not always align, underscoring the complexity of interpreting APN authority across diverse regulatory contexts.

**TABLE 2:** APN prescribing authority.

Prescribing authority	Respondent Agreement	Confirmed in comments	Countries
Established	Unanimous	Independent authority	Finland, Ireland, Netherlands, UK
Reported, but limited	Unanimous	Restrictions on prescribing authority	Estonia, France
Reported, not independent	Unanimous	Delegated authority	Lithuania
Reported, but emergent	Minority (1 of 3)	Prescribing not yet implemented, regulated or resourced	Austria, Belgium, Cyprus, Slovakia, Switzerland
Not reported	−	−	Albania, Belarus, Czechia, Denmark, Germany, Hungary, Iceland, (Kosovo), Kyrgyzstan, Luxembourg, Malta, Moldova, Norway, Portugal, Slovenia, Spain, Sweden, Tajikistan

Respondents clarified prescribing authority in the questionnaire free‐text comments, indicating the form of prescribing taken, hence further detail to the binary question. For the full data set, see Supplementary File S4.

### 5.4 APN‐Specific Regulation

Among the 29 countries reporting APN roles, 15 had established regulatory frameworks specifically governing this level of practice (Table 3). In all but one, regulatory authority resided with the national body responsible for nursing registration, and compliance was mandatory. The exception was Switzerland, which operated a voluntary registry administered by a professional APN association and underpinned by educational standards and clinical practice requirements.

**TABLE 3:** Distribution of regulatory organisation comparing the status of APN prescribing authority and APN‐specific regulation.

	Independent statutory body	Government	Hybrid	National Nursing Association	Other
APN regulation with independent prescribing	Ireland UK	Finland Netherlands			
APN regulation with limited prescribing	France	Estonia			
APN regulation without independent prescribing		Hungary Iceland Lithuania Luxembourg Norway Sweden Tajikistan	Switzerland Malta		
APN present, no regulation or prescribing		Austria Belarus Belgium Czechia Denmark Germany	Kyrgyzstan Moldova Slovakia Spain	Albania Cyprus (Kosovo) Portugal Slovenia	
No APN role		Bosnia and Herzegovina Russia Turkey Ukraine	Croatia Greece Italy Latvia	Bulgaria North Macedonia Poland Romania	Montenegro (unknown) Serbia (no regulation)

Partial regulatory arrangements were evident in a number of countries, reflecting incremental rather than comprehensive approaches to APN governance. Norway had established educational standards and title protection but had not yet granted prescribing authority. Finland and the United Kingdom regulated prescribing authority separately, independent of an advanced practice framework.

No country in this sample reported independent prescribing authority in the absence of APN‐specific regulation, although the reverse was common: Several countries, including Norway, had established regulatory frameworks without corresponding prescribing rights. A further pattern was observed in regulatory body type, with government‐led or legally mandated independent statutory oversight more common among countries with established prescribing authority, whereas models relying primarily on professional association governance corresponded with more constrained authority.

A clear readiness gap was evident, with 49 respondents indicating that existing regulatory structures in their country do not yet meet the requirements of APN, resulting from persistent barriers related to prescribing authority and professional autonomy. Only ten respondents considered current regulation to be adequate. These individuals fell into two distinct groups: first, those working within frameworks reporting comprehensive scope of practice and independent prescribing authority consistent with FPA, represented by all respondents from Ireland and the Netherlands; and second, those who believed that APNs should function exclusively under delegated authority. The remaining 38 respondents did not answer this item.

### 5.5 Interest‐Holder Opinions

Of the 97 respondents, 93 answered the Likert‐scale items. One regulator did not respond, noting a perceived conflict of interest in commenting on their own country's regulatory arrangements. Regardless of nationality or interest‐holder group, there was broad consensus among respondents in favour of regulating APN, with national standardisation seen as particularly important. Supplementary File 3 shows the mean scores and standard deviation for the Likert‐scale questions, detailing regulatory mechanisms. Participants also emphasised the importance of nursing leadership within government structures, such as the presence of a Government Chief Nursing Officer, in furthering APN regulation. Another pattern was evident in regulatory preferences related to practice authority: Acceptance of delegated or physician‐supervised authority was more common in Central, Eastern and Southern Europe, aligning with the earlier stages of APN role development in these regions. Overall, many countries appeared to mirror their existing nursing regulation systems, such that jurisdictions without features like registered nurse revalidation tended to be less supportive of introducing equivalent mechanisms for APNs.

## 6 Strengths and Limitations

Extending the questionnaire to WHO/Europe countries provided a comprehensive and region‐wide evaluation of progress towards strengthening regulatory systems and advancing the development of nursing roles. The survey demonstrated a high response rate, further reinforced by the active engagement of key interest‐holder groups, thereby enhancing the robustness and validity of the findings. However, responses per country often resulted in intra‐country disagreement; only a small number of jurisdictions showed complete alignment among interest‐holder groups regarding the distribution of regulatory responsibilities. These discrepancies likely reflect a combination of translation and definitional challenges, federal or regional variation, or possibly reflect the inherent complexity of nursing regulation. Similar reasons could also be the cause of the fluctuating agreement on APN characteristics, indicating that reported scopes of practice may function more as broad national estimates than as precise descriptions of legal practice boundaries.

Purposive, non‐probability sampling ensured participant expertise but may not represent the full range of perspectives within each country. The number of respondents per interest‐holder group also varied by country, affecting the comparability of classifications. Classifications rely on key informant report rather than independent legal verification and are presented as interest‐holders’ reported understanding rather than definitive statements of law.

This cross‐sectional, descriptive design can identify associations but not causation. No patient outcome or safety data were collected; therefore, this study is not positioned to draw conclusions regarding the safety of advanced practice models or regulatory mechanisms.

Data were collected between December 2023 and May 2024, and regulatory arrangements may have changed since.

The survey was intentionally designed without collecting detailed legal or jurisdictional breakdowns, and as such, finer regulatory distinctions fall outside the scope of this study and remain an important avenue for future research.

## 7 Discussion

Prescribing authority is an indicator of the comprehensive advanced practice model associated with the strongest improvements in health system performance (Htay and Whitehead 2021), and it emerged as the point at which regulatory attention concentrates. Regulation appears to function as a precondition on which prescribing authority depends, but having regulation in place does not by itself guarantee that authority follows. Notably, countries where prescribing authority was consistently and unanimously reported also had a comprehensive APN regulatory framework, raising the possibility that regulatory clarity reduces the disagreement otherwise found among interest‐holders describing APN scope of practice.

### 7.1 Reality of APN Implementation

ICN APN guidelines characterise advanced practice nursing as integrating nursing knowledge with expanded clinical competencies, underpinned by FPA. However, the low number of countries in which these features were reported indicates that, despite the high reported prevalence of APN roles, many have not developed to this level.

Respondents from Central and Western Asia reported that APNs operate within a relatively restricted scope of practice, viewing delegated responsibilities rather than independent practice as an appropriate model in their context. While any structured strengthening of nursing contributes positively to health system performance, many other respondents reported restrictions rooted in legal and regulatory boundaries and a misalignment between regulation and working reality for APNs. This aligns with Unsworth et al. (2024), who observed that roles frequently evolve ahead of the regulatory frameworks designed to govern them, leaving practice authority constrained by structures that no longer reflect clinical reality.

The persistence of APN roles even without specific regulation indicates strong profession‐led demand, frequently initiated as pragmatic responses to service pressure and access gaps. However, respondents who judged APN frameworks as commensurate with the clinical demands of the role reported working within a health system which incorporated comprehensive regulation and had a comprehensive scope of practice, notably Ireland and the Netherlands. Where professional APN regulation was incomplete or absent, the majority of respondents detailed issues in implementation, such as scope of practice variation in the United Kingdom or practice constraints, detailed in other countries.

This points to outdated professional regulation being associated with a substantial constraint on APN realising its full contribution.

### 7.2 Regulation of Advanced Practice

Countries that have established independent prescribing authority share notable regulatory characteristics, aligning with Stewart et al.’s (2021) guidance on the conditions needed to support independent prescribing. First, regulation is overseen either directly by government departments or by independent statutory bodies with a legal mandate to act on the government's behalf. These bodies tend to predominate over national nursing associations, likely because dismantling entrenched professional boundaries requires enforceable legal authority, something national nursing associations generally lack.

Second, these jurisdictions maintain a national framework providing cohesion for essential elements of advanced practice or high‐risk skills such as prescribing. This is consistent with Maier's (2015) finding that nationally regulated systems demonstrated greater standardisation and role clarity than decentralised or unregulated alternatives. Beyond ensuring regulatory consistency, this nationwide standard‐setting also functions as a socialisation process, familiarising interest‐holders with new roles and competencies (Dankers‐de Mari et al. 2023). This is clearly illustrated in the Dutch and Irish responses, where strong interest‐holder consensus across all aspects of APN roles and accompanying regulation reflected the normalisation associated with national frameworks.

Third, regulatory organisations in settings with diagnostic and prescribing authority commonly have a single mandate of public protection (National Council of State Boards of Nursing 2023). Where regulators combine public protection with professional promotion or trade union functions, a conflict of interest can arise (Benton et al. 2017). A focus on public protection is well‐suited to managing the augmented clinical risk that accompanies extended practice authority.

### 7.3 Scope of Practice and Clinical Risk

The extension of nurses’ scope of practice to incorporate competencies previously deemed the domain of medicine is rarely systematic, and role implementation is often incremental in nature, which seems to produce role variation, even where the same terminology is applied. History taking and physical examination are the most commonly reported aspects of advanced practice, likely because they support diagnostic processes without transferring full diagnostic responsibility, therefore allowing earlier APN integration into clinical workflows. In contrast, independent diagnosis and prescribing carry higher clinical risk, making political and managerial interest‐holders more cautious about their adoption. Willingness to grant diagnostic and prescribing authority therefore depends on confidence in APN competency standards, which professional regulation can help build among clinicians, employers and the public.

Debates around APN expansion reflect contrasting conceptualisations of healthcare itself. When health is framed as a service industry, efficiency and cost‐effectiveness dominate decision‐making and role expansion is viewed with suspicion as a quality‐diluting, cost‐reduction strategy (Mohamed et al. 2023). In contrast, healthcare, framed as a safety‐critical industry (Wears 2012), shifts regulatory implications as risk management and competency assurance become the primary drivers, meaning diagnostic and prescribing authority can be introduced as a controlled, quality‐focused intervention. The regulatory approach adopted by a health system therefore reflects, and reinforces, which framing prevails. This study's findings suggest that mandatory regulation with a singular public protection mandate operationalises the safety‐critical framing in practice, consistent with Duignan et al. (2025) risk‐based regulation argument. By explicitly prioritising patient safety, regulatory frameworks counter perceptions that advanced practice is merely a cheaper substitute for medical care, instead positioning APN as a strategic approach to enhancing healthcare delivery while maintaining or improving quality (De Leede‐Brunsveld et al. 2023). That this framing resonates beyond policy circles is evidenced by the consistently strong cross‐interest‐holder support in favour of APN regulation observed in this study (83.9%–96.8% agreement across individual mechanisms; Supplementary File S3), suggesting that regulatory reform was viewed by respondents as both important and welcomed by those closest to practice.

### 7.4 Integration of Regulatory Frameworks

A key finding is that APN‐specific regulation generally builds upon existing nursing regulatory frameworks. In all but one case, the same regulatory organisation responsible for nursing oversees APN regulation and mechanisms largely mirror those established for the broader profession. Where revalidation is absent for nurses, as in the Nordic countries, this is generally reflected in their APN regulatory approach. Notably, interest‐holders in jurisdictions without existing revalidation expressed less support for its introduction as an APN‐specific mechanism. This suggests that regulatory innovations are more readily adopted where foundational regulatory frameworks already exist, as interest‐holders may lack the contextual familiarity needed to evaluate or embrace new mechanisms.

These findings underscore the need for further research into the appropriateness and effectiveness of specific regulatory mechanisms for assuring APN competencies. Given that APNs assume responsibilities traditionally associated with physicians, examination of physician regulatory systems may help identify mechanisms adaptable for advanced practice nursing.

A further challenge arises in countries where nursing regulation is limited or absent, as the foundational infrastructure for credentialing, standard‐setting and oversight may be inadequate. The survey results indicate that established APN development is associated with nursing professionalisation underpinned by a robust professional regulatory base. Weaker regulatory foundations may leave the feasibility and sustainability of advanced practice uncertain, suggesting that investment in baseline nursing regulation may be an important step before further APN reform is attempted.

The findings highlight that APN regulation is also linked with interest‐holder familiarity with regulatory mechanisms and to alignment between regulatory expectations and levels of clinical responsibility. Where these foundations are stronger, pathways for the integration and expansion of APN appear more coherent, credible and trusted.

A final consideration is whether APN should be regulated as a distinct profession or whether oversight of specific high‐risk clinical skills, layered onto existing nursing regulation, is sufficient. Finland and the United Kingdom regulate nurse prescribing exclusively, without necessarily recognising it as an APN function. Ireland regulates both prescribing and advanced practice separately, with the distinguishing factor being a master's degree requirement consistent with ICN guidelines. The Netherlands similarly makes master's completion a prerequisite for its clinical competency register. No comparative evaluation has yet determined whether a professionally based or clinical‐skills–focused register more effectively mitigates clinical risk, leaving insufficient evidence to guide decision‐making on optimal regulatory structures.

## 8 Conclusion

This study's findings suggest that a significant barrier to APN's full contribution may be the persistence of outdated regulatory arrangements, rather than a lack of demand or capability. Mandatory, public‐protection‐focused regulation appears to support APNs extending their role to include the high patient‐risk tasks of independent diagnosis and prescribing. This pattern is consistent with Lehwaldt and Stoneman's (2024) argument that advanced practice models become both more feasible and safer to sustain under such regulatory conditions. Aligning regulation with contemporary clinical realities may help convert fragmented, practice‐based adaptations into a coherent strategy, supporting APN's system‐level value. Given the risks associated with unregulated scope‐of‐practice expansion identified elsewhere (Palmer et al. 2023), such alignment may also benefit patient safety.

Regulatory reform has consistently lagged behind clinical practice. Jurisdictions with empowered statutory regulators and nationally coherent frameworks appear to have greater capacity to modernise APN regulation than those relying on voluntary standards or variable implementation. Such transformation furthers global health priorities, aligning directly with the Universal Health Coverage agenda (United Nations 2019) and positioning health systems to leverage APN to strengthen service delivery and improve care accessibility.

## 9 Implications for Health and Nursing Policy

To maximise advanced practice contributions, countries seeking to develop advanced practice nursing could consider statutory, or legally mandated regulatory frameworks focused on publicprotection, while evaluating their suitability within the relevant legal, professional and health‐ system context. Where nursing regulatory foundations are weak or absent, strengthening this baseline may be an important step before meaningful APN regulatory reform can be attempted. Regulatory frameworks should reflect contemporary clinical realities rather than historical professional boundaries, ensuring legally defined scopes of practice align with extended competencies. Policymakers should also recognise that nursing leadership within government, such as a Government Chief Nursing Officer, could play an important role in driving this process. Given the current absence of comparative evidence on optimal regulatory models, research evaluating the effectiveness of different regulatory mechanisms in assuring APN competence and patient safety should be treated as a priority.

## Author Contributions

Caroline Bohlender: conceptualisation, methodology, investigation, formal analaysis, writing – original, review and edit, project administration. Paul Gill: writing – review and edit, supervision. Karen Bjoro: writing – review and edit, supervision. John Unsworth: writing – review and edit, supervision.

## Conflicts of Interest

The authors declare no conflicts of interest.

## Funding

The authors have nothing to report.

## Supporting information




**Supplementary File 1**. Questionnaire used in research project into regulation and Advanced Practice Nursing (APN) development.

**Supplementary Table 2**. Scope of practice reported by respondents for Advanced Practice Nurses, by country.

**Supplementary File 3**. Importance of regulatory mechanisms and factors influencing the development of Advanced Practice Nursing.

**Supplementary File 4**. Questionnaire responses and open text comments for categorisation of prescribing authority.
