# Supplementary material for: Advanced Practice Nursing Development Within Diverse Regulatory Landscapes: A Descriptive Survey of European Countries
Source: Int Nurs Rev. 2026 Oct 12;73(4):e70250. doi: 10.1111/inr.70250 (PMC13656334; doi:10.1111/inr.70250)
Supplement: Supplementary file 1 [file INR-73-0-s001.docx]

**Supplementary File 1 – Questionnaire used in research project into regulation and Advanced Practice Nursing (APN) development.**

This questionnaire was designed to identify 1) regulatory organisations and mechanisms for nursing and APN, 2) APN scope of practice and 3) expert opinion of key topics relating to APN development.

Regulation and Advanced Practice

# Introduction and Consent

Thank you for responding to the invitation to take part in this study. Your participation is valuable and your expertise important.

**Participant Information** Before you decide whether to participate, it is important for you to understand why the research is being carried out and what it will involve. Please take time to read the information carefully, discuss it with others and ask any questions you may have.

The aim of this study is to explore the contribution of national regulatory systems on the development of advanced nursing practice (ANP) roles within Europe. The objective is to establish whether a certain typography or characteristics of a regulatory structure can facilitate or hinder post-basic nursing practice development.

No, there is no obligation to take part. If you decide to take part, you will be asked to sign a consent form. You are free at any time to withdraw from the study, and do not have to give a reason. If you do proceed, you will be asked to complete a short questionnaire about regulation and advanced nursing practice. This should take 15-20 minutes to complete.

The findings of this study will seek to provide recommendations based on best regulatory practice to support developments in advanced nursing practice as required. Your input will be valued and crucial to provide information on which to base any recommendations. Other than you time, there are not disadvantages to taking part in this questionnaire.

All data collected in this study will be fully anonymised using numerical coding to maintain confidentiality. Only the researcher will have access to any identifiable information which will be kept separate from any data that can identify you, unless you have specifically given permission for your name to be used. All data will be stored on a password protected computer in accordance with university guidelines and the Data Protection Act (2018). At no point will your personal information or data be revealed.

The results of the study will be shared with key stakeholders, including WHO Europe

office, ICN and wider community via conference presentation and journal publication. If you would like to be kept informed of the results of this study, email XXXXXX

XXXXX is funding the study as part of a studentship programme and has full ethical approval from XXXXXXXX ethics committee.

If you have any questions please email XXXXXXXXX who will be happy to answer them.

If you do not wish to proceed

Decline and leave this questionnaire

**Consent** If you are happy to proceed and have no further questions, please indicate your agreement with the statements below, by selecting each option.  *Required*

Please select at least 5 answer(s).

I have carefully read and understood the Participant Information presented above.

I have had an opportunity to ask questions and discuss this study and I have received satisfactory answers.

I understand I am free to withdraw from the study at any time, without having to give a reason for withdrawing, and without prejudice.

I understand that if I would like to receive feedback on the overall results of the study I must contact the researcher at [XXXXXXXXX](mailto:caroline.bohlender@northumbria.ac.uk)

I agree to take part in this study.

Name:

# Question 1 - Background

Qu 1.1 - In which country do you work?

Qu 1.2 - What is your job title?

Qu 1.3 - What is your gender identification?

Male Female

Prefer not to say

Qu 1.4 - What is your professional background?

Nurse Doctor Midwife

Other (please specify)

If you selected Other, please specify:

Qu 1.5 - How long have you been qualified?

0-5 years

6-10 years

11-20 years

21 + years

# Question 2 - National Regulatory System

Qu 2.1 - Which organization regulates nursing practice in your country?  *Required*

Government department National nursing organization

Independent statutory body such as a Nursing Council Combination

Other Don't know

If you selected Other, please specify;

Qu 2.2 - What do they regulate? Please select all which apply.

Professional Register Title protection

Revalidation / Continuous Professional Development Education standards at entry level

Scope of practice Disciplinary procedures Other

Don't know

If you selected Other, please specify:

Qu 2.3 - Does the organisation regulate;

Nurses Midwives Doctors

Healthcare Professionals eg physiotherapists, pharmacists Other

If you selected Other, please specify:

# Question 3 - Advanced Nursing Practice

Advanced Nursing Practice as defined by the ICN is "a field of nursing that extends and expands the boundaries of nursing’s scope of practice, contributes to nursing knowledge and promotes advancement of the profession". Advanced Nursing Practice will be used here as a term which encompasses all roles, regardless of title used.

Qu 3 - Are there advanced nursing practice roles in your country?  *Required*

Yes (to Question 4) No (to Question 8) Don't Know

# Question 4 - Advanced Nursing Practice Roles

Qu 4.1 - What are the job titles of these roles?  *Required*

Advanced Nurse Practitioner Advanced Clinical Practitioner Clinical Nurse Specialist Nurse Consultant

Other

If you selected Other, please specify:

Qu 4.2 - What is the scope of practice for these roles; please indicate all which apply 

*Required*

Clinical

Leadership / Management Education

Research Other

If you selected Other, please specify:

Qu 4.3 - In relation to clinical practice, do these roles involve; please tick all which apply.

Medical history taking Physical examination

Ordering diagnostic investigations eg X-ray

Complex treatment procedures eg surgery / anaesthetics Prescribing

Referral to other specialties / professionals Other

If you selected Other, please specify:

Qu 4.4 - What are the limitations, if any, to the scope of practice of an advanced nursing practitioner?

Qu 4.5 - Is there regulation for advanced nursing practice roles in addition to basic level registration?

Yes (to Question 5) No (to Question 9)

# Question 5 - Advanced Nursing Practice Regulation

Question 5 - Is the regulator the same as the entry level registration?

Yes, it is the same organization (to Question 6) No, it is a different organization (to Question 7)

# Question 6 - Same Regulatory System as Initial Registration

Qu 6.1 - Is this advanced practice regulation mandatory (required to practice at this level) or voluntary (optional)?

Mandatory Voluntary Don't Know

Qu 6.2 - What is regulated?

Title protection Register

Educational standards for advanced practice Scope of practice

Discipline procedures Other

Don't know

If you selected Other, please specify:

# Question 7 - Alternative Advanced Nursing Practice Regulation

Qu 7.1 - Who is responsible for advanced nursing practice regulation and how does it differ from the entry level registration?

Qu 7.2 - Is this advanced nursing practice regulation mandatory (required to practice at this level) or voluntary (optional)?

Mandatory Voluntary Don't Know

Qu 7.3 - What is regulated?

Title protection Register

Educational standards for advanced practice Scope of practice

Discipline procedures Other

Don't know

If you selected Other, please specify:

# Question 8 - No Advanced Nursing Practice

Qu 8.1 - Why is this role not present? Please indicate all which apply.

Role unknown Role not needed

Does not fit with healthcare system structures Opposition to the role

Legislation / Regulation parameters do not allow for advanced practice Other

If you selected Other, please specify:

Qu 8.2 - Are there plans to incorporate this role into the healthcare system in the future?

Yes No

# Question 9 - Regulatory Elements

Qu 9.1 - Consider each statement on title protection and decide on the extent to which you agree or disagree with each statement. Please select one answer per row or leave blank if don't know.

Please don't select more than 1 answer(s) per row.

|  | Strongly agree | Agree | Undecided | Disagree | Strongly Disagree |
| --- | --- | --- | --- | --- | --- |
| The title of nurse should be protected by law. |  |  |  |  |  |
| The regulator should maintain a register of nurses licensed to practice. |  |  |  |  |  |
| The regulator's nursing register should only relate to initial qualification as a registered nurse. |  |  |  |  |  |
| Specialisms and other qualifications should also be recorded on the register. |  |  |  |  |  |
| Advanced Nursing Practitioner qualifications should be recorded on the register. |  |  |  |  |  |

| Maintaining a license to practice should require nurses to maintain their knowledge and skills through continuing education. |  |  |  |  |  |
| --- | --- | --- | --- | --- | --- |
| Maintaining a license to practice should require nurses to maintain the knowledge and skills through a specified number of practice hours. |  |  |  |  |  |

Qu 9.2 - Consider each statement and decide on the extent to which you agree or disagree with each statement. Please select one answer per row or leave blank if don't know.

Please don't select more than 1 answer(s) per row.

|  | Strongly agree | Agree | Undecided | Disagree | Strongly Disagree |
| --- | --- | --- | --- | --- | --- |
| The regulator should specify the scope of practice. |  |  |  |  |  |
| The scope of professional practice should set out the duties of practice. |  |  |  |  |  |

| The scope of practice should be defined by the nurse's training and competence. |  |  |  |  |  |
| --- | --- | --- | --- | --- | --- |

Qu 9.3 - Consider each statement and decide on the extent to which you agree or disagree with each statement. Please select one answer per row or leave blank if don't know.

Please don't select more than 1 answer(s) per row.

|  | Strongly agree | Agree | Undecided | Disagree | Strongly Disagree |
| --- | --- | --- | --- | --- | --- |
| The regulator should set the academic level eg. degree for qualifications leading to initial registration as a nurse. |  |  |  |  |  |
| The competencies and proficiencies for practice should be defined. |  |  |  |  |  |
| The regulator should set general standards eg, programme length, academic level, content etc. |  |  |  |  |  |
| Universities should define programme specifications. |  |  |  |  |  |

# Question 10 - Regulatory Elements continued

Qu 10.1 - Consider each statement and decide on the extent to which you agree or disagree with each statement. Please select one answer per row or leave blank if don't know.

Please don't select more than 1 answer(s) per row.

|  | Strongly agree | Agree | Undecided | Disagree | Strongly Disagree |
| --- | --- | --- | --- | --- | --- |
| The regulator should set standards for qualifications beyond initial registration eg specialisms. |  |  |  |  |  |
| The regulator should set standards for Advanced Nursing Practice. |  |  |  |  |  |
| The regulator should set standards for prescribing. |  |  |  |  |  |

Qu 10.2 - Consider each statement and decide on the extent to which you agree or disagree with each statement. Please select one answer per row or leave blank if don't know.

Please don't select more than 1 answer(s) per row.

|  | Strongly agree | Agree | Undecided | Disagree | Strongly Disagree |
| --- | --- | --- | --- | --- | --- |

| The regulator should set standards for aspects of practice eg, medicines administration. |  |  |  |  |  |
| --- | --- | --- | --- | --- | --- |

Qu 10.3 - Consider each statement and decide on the extent to which you agree or disagree with each statement. Please select one answer per row or leave blank if don't know.

Please don't select more than 1 answer(s) per row.

|  | Strongly agree | Agree | Undecided | Disagree | Strongly Disagree |
| --- | --- | --- | --- | --- | --- |
| The regulator should develop a code of conduct and ethics. |  |  |  |  |  |
| Nurses who fail to adhere to the standards of the code of conduct should be sanctioned by the regulator eg. removal of license to practice. |  |  |  |  |  |

# Question 11 - Drivers of Advanced Nursing Practice

Qu 11 - Consider each statement and decide on the extent to which you think these factors are important in influencing development of advanced nursing practice in your country. Please select one answer per row or leave blank if don't know.

Please don't select more than 1 answer(s) per row.

|  | Extremely important | Important | Undecided | Unimportant | Extremely unimportant |
| --- | --- | --- | --- | --- | --- |
| Political will and desire is needed for advanced nursing practice development. |  |  |  |  |  |
| Opinion and acceptance of other professions in the multidisciplinary team eg, Medicine. |  |  |  |  |  |
| The need to meet Universal Health Coverage goals. |  |  |  |  |  |
| Service delivery pressures and / or access issues. |  |  |  |  |  |
| Shortage of staff in other professions. |  |  |  |  |  |
| Need to provide more cost efficient healthcare. |  |  |  |  |  |

# Question 12 - Aspects of Advanced Nursing Practice.

Qu 12.1 - Consider each aspect within scope of practice and decide on the extent to which you agree or diasagree that these areas need to be part of advanced nursing practice. Please select one answer per row or leave blank if don't know.

Please don't select more than 1 answer(s) per row.

|  | Strongly agree | Agree | Undecided | Disagree | Strongly disagree |
| --- | --- | --- | --- | --- | --- |
| Diagnostic Skills, eg. medical history taking, physical examination |  |  |  |  |  |
| Diagnostic intervention authority, eg. ordering X-ray |  |  |  |  |  |
| Complex treatment procedures , eg. surgery / anaesthetics |  |  |  |  |  |
| Prescribing |  |  |  |  |  |
| Referral authority to other specialties or professionals |  |  |  |  |  |

Qu 12.2 - Consider each statement and decide on the extent to which you agree or disagree that these aspects are essential to development of advanced nursing practice roles. Please select one answer per row or leave blank if don't know.

Please don't select more than 1 answer(s) per row.

|  | Strongly agree | Agree | Undecided | Disagree | Strongly disagree |
| --- | --- | --- | --- | --- | --- |

| The healthcare system needs advanced nursing practitioner roles. |  |  |  |  |  |
| --- | --- | --- | --- | --- | --- |
| Supervision by a doctor is needed for an advanced nursing practitioner to do role safely. |  |  |  |  |  |
| An advanced nursing practitioner should be able to work autonomously. |  |  |  |  |  |
| Advanced nursing practice needs to include research evidence. |  |  |  |  |  |
| Implementation of new advanced nursing practice roles needs strong leadership. |  |  |  |  |  |
| Advanced nursing practice needs strong nursing leadership at policy making level. |  |  |  |  |  |
| Advanced nursing practice training and credentialing should be standardized nationally. |  |  |  |  |  |

| Advanced nursing practice should be responsive to local healthcare needs. |  |  |  |  |  |
| --- | --- | --- | --- | --- | --- |

# Final page

Heart felt thanks for taking the time to complete this questionnaire. Your information and opinions are important to the value of this research.

The results from the survey will be shared as soon as analysis is completed.
