# Supplementary material for: Advanced Practice Nursing Development Within Diverse Regulatory Landscapes: A Descriptive Survey of European Countries
Source: Int Nurs Rev. 2026 Oct 12;73(4):e70250. doi: 10.1111/inr.70250 (PMC13656334; doi:10.1111/inr.70250)
Supplement: Supplementary file 2 [file INR-73-0-s002.docx]

**Supplementary File 3: Importance of regulatory mechanisms and factors influencing the development of Advanced Practice Nursing**

Likert scale responses for items relating to APN development, ordered by the proportion of respondents rating each item as important.

____________________________________________________________________________________________________________________________________________________________

Survey item	Extremely	Important (4)	Undecided (3)	Unimportant (2)	Extremely	Mean

*How important*	Important (5)	Unimportant (1)

____________________________________________________________________________________________________________________________________________________________

is it that APNs are	n = 66 (71.0%)	n = 12 (12.9%)	n = 9 (9.7%)	n = 4 (4.3%)	n = 2 (2.2%)	4.5

Registered?

is it that APN should be a	n = 75 (80.6%)	n = 15 (16.1%)	n = 3 (3.2%)	n = 0 (0%)	n = 0 (0%)	4.8

Protected title?

is Masters as entry to	n = 79 (84.9%)	n = 10 (10.8%)	n = 2 (2.2%)	n = 2 (2.2%)	n = 0 (0%)	4.8

Practice?

is revalidation?	n = 65 (69.8%)	n = 21(22.6%)	n = 6 (6.4%)	n = 1 (1.1%)	n = 0 (0%)	4.6

are advanced Practice	n = 50 (53.7%)	n = 35 (37.7%)	n = 6 (6.4%)	n = 1 (1.1%)	n = 1 (1.1%)	4.4

roles to the healthcare

system in your country?

is it to have national	n = 65 (69.9%)	n = 25 (26.9%)	n = 3 (3.2%)	n = 0 (0%)	n = 0 (0%)	4.7

standardisation for

advanced practice roles?

is it to have standardisation	n = 49 (52.7%)	n = 30 (32.3%)	n = 11 (11.8%)	n = 3 (3.2%)	n = 0 (0%)	4.2

of advanced practice roles

throughout Europe?

is it that advanced practice	n = 58 (62.4%)	n = 32 (34.4%)	n = 3 (3.2%)	n = 0 (0%)	n = 0 (0%)	4.6

nurses to be involved in

research?

is it to have strong	n = 70 (75.3%)	n = 19 (20.4%)	n = 3 (3.2%)	n =1 (1.1%)	n = 0 (0%)	4.7

professional leadership at

a local level to facilitate

APN implementation?

is it for APNs to have	n = 70 (75.3%)	n = 19 (20.4%)	n = 4 (4.3%)	n = 0 (0%)	n = 0 (0%)	4.6

representation at policy

making level?

is it to have a national	n = 61 (65.6%)	n = 19 (20.4%)	n = 12 (12.9%)	n = 1 (1.1%)	n = 0 (0%)	4.5

government chief nursing

officer in place for advanced

practice to develop?

is the acceptance of the	n = 50 (53.7%)	n = 34 (36.6%)	n = 7 (7.5%)	n = 1 (1.1%)	n = 1 (1.1%)	4.4

medical profession to enable

APN development?

is the acceptance of other	n = 45 (48.3%)	n = 34 (36.6%)	n = 9 (9.7%)	n = 5 (5.4%)	n = 0 (0%)	4.3

healthcare professionals to

enable APN development?
