# Supplementary material for: Advanced Practice Nursing Development Within Diverse Regulatory Landscapes: A Descriptive Survey of European Countries
Source: Int Nurs Rev. 2026 Oct 12;73(4):e70250. doi: 10.1111/inr.70250 (PMC13656334; doi:10.1111/inr.70250)
Supplement: Supplementary file 3 [file INR-73-0-s003.docx]

**Supplementary File 4. Questionnaire responses and open text comments for categorisation of prescribing authority.**

Questionnaire responses to inclusion of prescribing within the APNs scope of practice. All countries were included which returned at lease one positive response. The free text comments provided by respondents allowed for a more detailed classification in Table 2.

| **Country** | **Prescribing – agreement of respondents and inter-holder group when less than 3** | **Open comments detailing authority or limitations on scope of practice from all country respondents**  **(respondents own words)** | **Interpretation of responses for prescribing authority** |
| --- | --- | --- | --- |
| Estonia | 2/2 = Association representative and Regulator | Today, APN rights are relatively limited, but we hope that new rights will enter into force in 2024: the right to prescribe medical devices, extended prescription rights, e-consultations, referral to other specialties / professionals, admission and discharge from hospital and other services | Prescribing, reported but limited authority – at time of completion |
| Finland | 3/3 | Only nurse prescribers allowed to prescribe, not part of role and education separate.  Prescription separate to MSc programme  Prescribing is a separate course but comes with authority | Established prescribing authority |
| Ireland | 3/3 | Individual SoP depends on what trained to do but all APNs are prescribers. Have full practice authority to complete full episode of patient care  Person dependent and on roles. Need to feel comfortable with all procedures and this can vary  Scope determined by education and authority agreed within practitioners' health service setting. Competence capability | Established prescribing authority |
| Lithuania | 2/2 = Association representative and Educator | Employers not expanding role and do not intend to use role  New role and SoP widening  Medical prescription done my Dr | Reported but not independent |
| UK | 3/3 | Full practice authority, autonomous and level, not title, is the issue  SoP individual nurse education, experience and competence. AP reg under review  No restrictions but individual SOP meaning not all APNs have same SOP. | Established prescribing authority |
| France | 2/2 = Association representative and Educator | Not 1st line [patients] by GP referral - Dx [Diagnosis] and Rx [treatment] initial by GP.  SoP set to evolve | Prescribing, reported but limited authority |
| Netherlands | 2/2 = Association representative and Educator | Only limitations to scope of practice are certifying death, mental health sectioning | Established prescribing authority |
| Slovakia | 1/3 - Association representative | Roles are not yet implemented  Limitations include  Lack training, Medicine is barrier, Lack acceptance hospitals, regulation for competencies  Medical organisations do not want to accept ANP  Nurse shortage, competencies not defined | Prescribing reported, but emergent |
| Belgium | 1/3 Association representative | Legislation = acts by Drs - hope to change  Consultations done under Dr supervision  Specific roles not yet defined by law, but will inc all roles  No financial possibility to work independently  All acts supervised by Dr and payments to Dr  Law was passed a few years ago, are forming them but role not defined enough  Recognition of APN title in law but no usable details for practice | Prescribing reported, but emergent |
| Cyprus | 1/3  Association representative | All stakeholders still in discussions about implementation of role  No law regulation for APN but training  Trained APN but not active due to lack of regulation | Prescribing reported, but emergent |
| Austria | 1/3  Association representative | Missing defined SoP  Lack APN in legal framework of nursing, so cannot task share  Lacks Professional law | Prescribing reported, but emergent |
| Switzerland | 1/3  Educator | Indiv SoP based on learning and competencies. Financial limitations, not reimbursed, so not work as can as not paid  Depends on workplace and related physician specialists  Jurisdiction not allow full SoP | Prescribing reported, but emergent |
