# Supplementary material for: Advanced Practice Nursing Development Within Diverse Regulatory Landscapes: A Descriptive Survey of European Countries
Source: Int Nurs Rev. 2026 Oct 12;73(4):e70250. doi: 10.1111/inr.70250 (PMC13656334; doi:10.1111/inr.70250)
Supplement: Supplementary file 4 [file INR-73-0-s004.docx]

**Supplementary Table 2: Scope of practice reported by respondents for Advanced Practice Nurses, by country.**

This table shows the responses for each country regarding the clinical skill set needed to complete a full episode of patient care. Numbers of respondents per country are given, to a maximum of three, to show level of agreement.

| **Country** | **n** | **Perform a physical examination** | **Take a medical history** | **Order diagnostic tests** | **Prescribe medications** | **Refer to other specialists** | **Admit and discharge patients to hospital** |
| --- | --- | --- | --- | --- | --- | --- | --- |
| ***Central Asia*** | | | | | | | |
| Kyrgyzstan | 3 |  3/3 |  3/3 |  1/3 |  0/3 |  3/3 |  0/3 |
| Tajikistan | 3 |  2/3 |  2/3 |  1/3 |  0/3 |  2/3 |  1/3 |
| ***Eastern Europe*** | | | | | | | |
| Belarus | 2 |  0/2 |  1/2 |  0/2 |  0/2 |  1/2 |  0/2 |
| Czech Republic | 2 |  1/2 |  1/2 |  1/2 |  0/2 |  0/2 |  1/2 |
| Hungary | 1 |  1/1 |  1/1 |  1/1 |  0/1 |  1/1 |  0/1 |
| Moldova | 3 |  2/3 |  2/3 |  0/3 |  0/3 |  2/3 |  0/3 |
| Slovakia | 3 |  3/3 |  1/3 |  2/3 |  1/3 |  1/3 |  2/3 |
| ***Northern Europe*** | | | | | | | |
| Denmark | 1 |  0/1 |  0/1 |  0/1 |  0/1 |  1/1 |  0/1 |
| Estonia | 2 |  2/2 |  2/2 |  2/2 |  2/2 |  0/2 |  0/2 |
| Finland | 3 |  3/3 |  3/3 |  3/3 |  3/3 |  3/3 |  1/3 |
| Iceland | 3 |  2/3 |  2/3 |  1/3 |  0/3 |  2/3 |  1/3 |
| Republic of Ireland | 3 |  3/3 |  3/3 |  3/3 |  3/3 |  3/3 |  3/3 |
| Lithuania | 2 |  0/2 |  2/2 |  1/2 |  1/2 |  2/2 |  0/2 |
| Norway | 3 |  3/3 |  3/3 |  0/3 |  0/3 |  0/3 |  0/3 |
| Sweden | 2 |  2/2 |  2/2 |  2/2 |  0/2 |  2/2 |  2/2 |
| United Kingdom | 3 |  3/3 |  3/3 |  3/3 |  3/3 |  3/3 |  3/3 |
| ***Southern Europe*** | | | | | | | |
| Albania | 3 |  0/3 |  0/3 |  0/3 |  0/3 | 0/3 | 0/3 |
| Malta | 3 | 1/3 | 0/3 | 1/3 | 0/3 | 1/3 | 0/3 |
| Portugal | 2 | 2/2 | 1/2 | 0/2 | 0/2 | 2/2 | 0/2 |
| (Kosovo) | 2 | 0/2 | 0/2 | 0/2 | 0/2 | 0/2 | 0/2 |
| Slovenia | 3 | 1/3 | 1/3 | 1/3 | 0/3 | 0/3 | 0/3 |
| Spain | 2 | 1/2 | 1/2 | 0/2 | 0/2 | 0/2 | 0/2 |
| ***Western Asia*** | | | | | | | |
| Cyprus | 3 | 2/3 | 2/3 | 1/3 | 1/3 | 2/3 | 1/3 |
| ***Western Europe*** | | | | | | | |
| Austria | 3 | 0/3 | 1/3 | 0/3 | 1/3 | 2/3 | 1/3 |
| Belgium | 3 | 2/3 | 2/3 | 0/3 | 1/3 | 3/3 | 0/3 |
| France | 2 | 2/2 | 2/2 | 2/2 | 2/2 | 2/2 | 1/2 |
| Germany | 3 | 1/3 | 0/3 | 1/3 | 0/3 | 1/3 | 1/3 |
| Luxembourg | 1 | 1/1 | 1/1 | 1/1 | 0/1 | 1/1 | 1/1 |
| Netherlands | 2 | 2/2 | 2/2 | 2/2 | 2/2 | 2/2 | 2/2 |
| Switzerland | 3 | 2/3 | 2/3 | 1/3 | 1/3 | 0/3 | 0/3 |

*Note: The fraction and shaded icon in each cell show the number of respondents from that country who reported the characteristic, out of the total number of respondents from that country (n). An unshaded circle indicates that no respondent from that country reported the characteristic. Respondents per country comprised professional regulators, nurse educators and national nursing association representatives.*
